# Sustainable, Recyclable, and Bench-Stable Catalytic System for Synthesis of Poly(ester-*b*-carbonate)

**DOI:** 10.1021/cbe.4c00064

**Published:** 2024-06-03

**Authors:** Yifan Jia, Bokun Li, Yifei Sun, Chenyang Hu, Xiang Li, Shunjie Liu, Xianhong Wang, Xuan Pang, Xuesi Chen

**Affiliations:** † Key Laboratory of Polymer Ecomaterials, Changchun Institute of Applied Chemistry, Chinese Academy of Sciences, 5625 Renmin Street, Changchun 130022, People’s Republic of China; ‡ School of Applied Chemistry and Engineering, University of Science and Technology of China, Hefei 230026, People’s Republic of China

**Keywords:** supported catalyst, multiblock copolymer, green metal, lactide, CO_2_-based copolymer

## Abstract

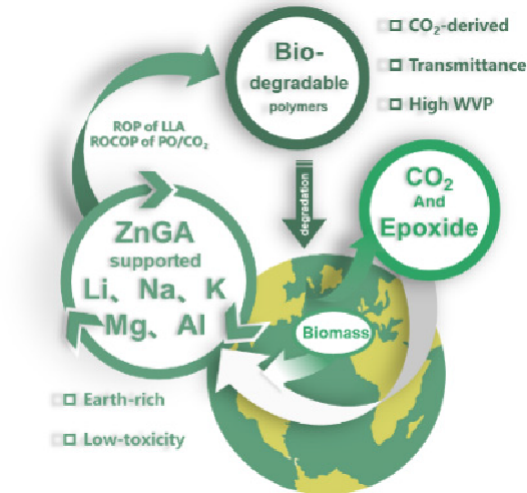

Transferring abundant, inexpensive, and nontoxic carbon dioxide (CO_2_) into biodegradable polymers is one of the ideal ways to promote sustainable development. Although a great deal of preeminent researches has been reported in the last decade, including well-designed organometallic complexes, Lewis pairs, etc. The moisture- and air-sensitive nature of these extensively used catalysts preclude their use in industrial applications. Herein, we report a novel stable catalyst system of commercial zinc glutarate (ZnGA) with a supported metal for the synthesis of poly(ester-*b*-carbonate). The special supported microstructure facilitates efficient polymerizations via a plausible heterometal coordination mechanism. Notably, the resulted biodegradable CO_2_-based copolymer showed strong tensile strength (>40 MPa), improved elongation (45% versus 7%), excellent transmittance, and low water vapor permeability (WVP) (1.7 × 10^–11^ g m^–1^ s^–1^ Pa^–1^). Moreover, the supported ZnGA catalyst is recyclable, and its simple and low-cost preparation process is compatible with the manufacturing and processing methods of the existing infrastructure.

## Introduction

One of the urgent global challenges is to replace the widely applied petroleum-derived and environmentally long-lasting polymers with sustainable alternatives.^1^ As a greenhouse gas, carbon dioxide (CO_2_) is harmful to the climate, but, at the same time, it is abundant and inexpensive. Therefore, it would be ideal to prepare biodegradable polymers by exploiting CO_2_ as a raw material.^2−8^ Since the pioneering work of Inoue in 1969, ring-opening copolymerization (ROCOP) of epoxide and CO_2_ has been intensively studied.^5,9−12^ At present, industrial aliphatic CO_2_-based alternating polycarbonate is mainly used in the polyurethane manufacture as the low-molar-mass polyol parts for its special mechanical properties and thermal properties.^13,14^ However, considering that polyurethane could not be 100% biodegraded, it is hard to fully achieve the above sustainable strategy.^15^ Hence, biodegradable segments are introduced to replace the rigid segments of polyurethane for more sustainable polymers. These revolutionary synthesis methods involve the tandem polymerization strategy and the chain component design.^16−20^ For the synthesis of biodegradable rigid segments, two controllable polymerizations are widely applied: lactide (LA) ring-opening polymerization (ROP) and phthalic anhydride (PA)/propylene oxide (PO) ROCOP.^21,22^ Although numerous catalysts have been already reported for the ROP of LA or the ROCOP of PO/CO_2_ individually, only a few of them showed activity for both polymerization processes in parallel.^23−26^

The development of novel catalyst systems, e.g., metal-based catalysts, Lewis pairs, and organocatalysts, has made great strides in the aforementioned designing of sustainable polymers, which are favorable for approaching the synthesis of desirable polymers.^16,19,27−36^ Pioneering work about metal-based catalysts from the groups of Coates, Nozaki, and Williams, amongst others, demonstrated the significant potential of dinuclear catalysts in CO_2_/epoxide ROCOP, the key of these proposed mechanistic hypotheses being that two metal centers at optimum distances could coordinate and catalyze the copolymerization with significantly enhanced activity.^2,20,23,31,37−40^ Furthermore, we and other groups have reported switchable polymerization, some of which showed better performances in tandem polymerization strategy.^16,19,20,25,29,41^ In terms of nonmetal catalysts, Li and other researchers reported multiblock poly(ester-*b*-carbonate) copolymers through the orthogonal copolymerization of epoxides/CO_2_ and ROCOP of CHO/PA with the help of Lewis pairs.^42−46^ Meanwhile, similar Lewis pair also were reported to successfully synthesize multiblock copolymers by sequential ROCOP of cyclohexene oxide (CHO)/PA and ROP of LLA.^46,47^

Nevertheless, the industrial adoption of these above copolymers was developing slowly, despite the numerous reports of excellent catalysts. This is primarily due to the fact that most of these well-designed catalysts are costly, as well as being water- and oxygen-sensitive, which cannot be aligned with industrial processing needs.^48−50^ Consequently, when researchers are dedicating themselves to synthesizing more sustainable and high-performance polymers for industrial application, they should conform the following principle. First, these new polymer manufacturing and processing methods should be compatible with the existing infrastructure. Second, the cost of catalysts must be economic; otherwise, it will become an industrial barrier. Finally, for the catalyst containing metal, the catalyst residue must not be too high, which will lead to a cost increase and ruin the ultimate material properties. Generally, the random or block polycarbonate polyesters reported were synthesized through homogeneous catalysts, and it is hard to match the above principles. Meanwhile, the catalysts in the heterogeneous field are also challenging to achieve the tandem of polymerization reactions.^51,52^ However, in our earlier works, a heterogeneous ternary catalyst system that consisted of SalenCo^III^, zinc glutarate (ZnGA), and PPNCl, which are stable, low-, and active, showed attractive features in the synthesis of multiblock poly(ester-*b*-carbonate) copolymers.^53,54^ Hence, we choose the traditional industrial catalyst ZnGA and its upgradation form in this research.^55^

**Scheme 1:**
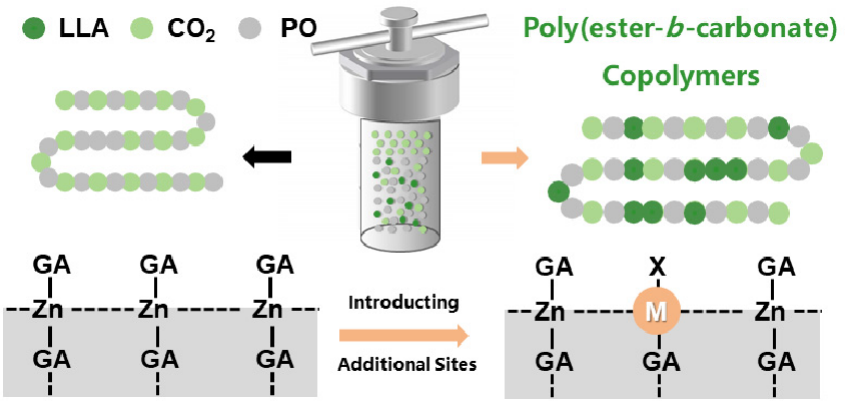
Application of Main Group Metal-Supported ZnGA in the Synthesis of Sustainable Polymers

With the appetite for sustainability continuing to increase throughout the polymerization process in mind, the design of catalyst should preferably meet the demand of being inexpensive and efficient, as well as having low toxicity.^6,56−58^ According to earlier research, taking into account the existing moisture- and air-stable and economical advantages of ZnGA, we propose a new strategy of catalyst design: using ZnGA as the carrier and the main group metal salts as the active metal surface sites to build a green, stable, and recyclable industrial catalytic system (Scheme 1). The supported ZnGA can provide biodegradable and waterproof CO_2_-based plastic materials with outstanding transmission and expected mechanical properties. In the future, these materials may substitute petrochemical general plastics and be utilized in high-growth fields such as packing and medicine.

## Results and Discussion

**Figure 1:**
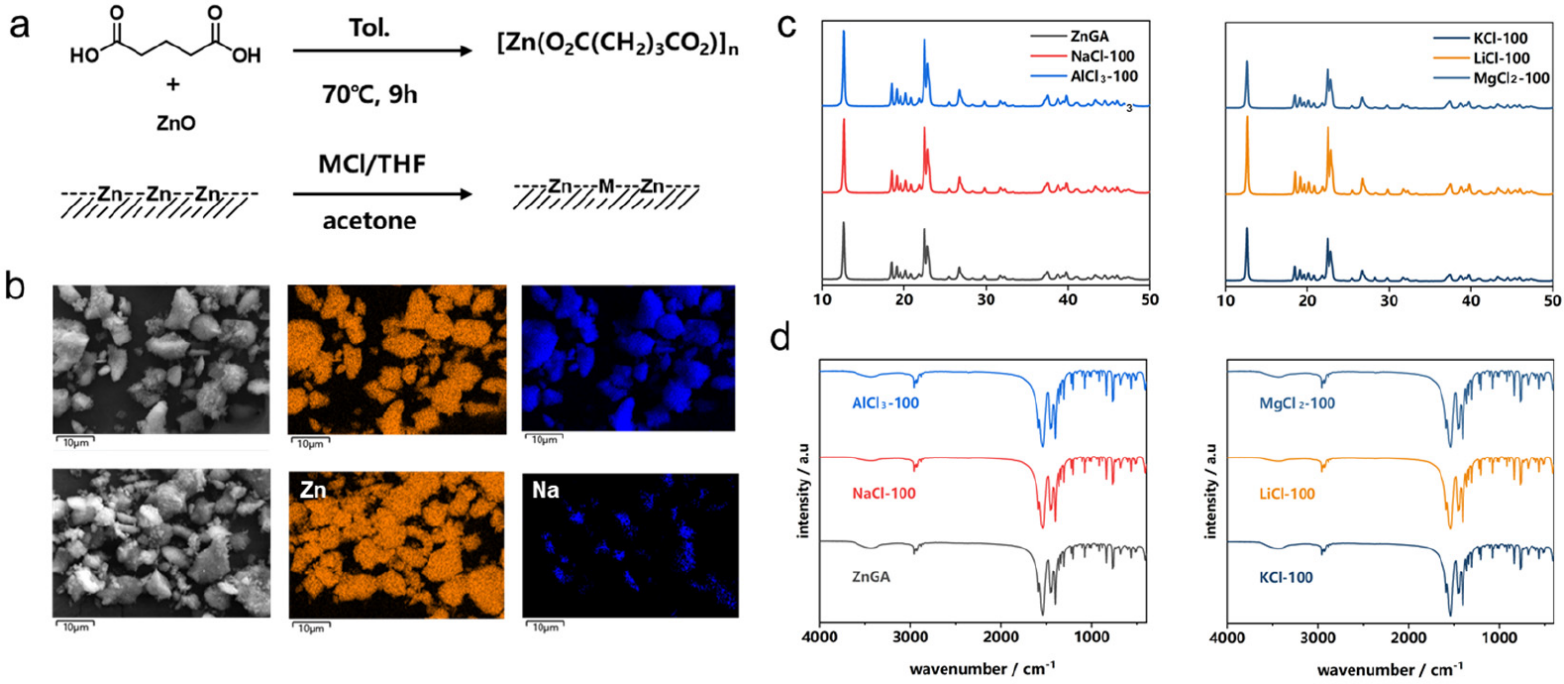
(a) Schematic diagram of the synthesis of supported catalysts. (b) SEM-EDS images of NaCl-100 (top) and its blends in the same proportion (zinc shown in yellow, sodium shown in blue). (c) PXRD images of ZnGA, NaCl-100, LiCl-100, KCl-100, MgCl_2_-100, and AlCl_3_-100. (d) FTIR images of ZnGA, NaCl-100, LiCl-100, KCl-100, MgCl_2_-100, and AlCl_3_-100. The resulted catalyst is named according to the type and amount of metal salt used, such as NaCl-100, which means that the type of metal salt used in the loading process is NaCl, and the proportion used is 1% of the ZnGA load.

The heterogeneous ZnGA, as a commercial catalyst, is attractive because of its low cost, low toxicity, and high activity, and it has been studied in the copolymerization of epoxides and CO_2_ for more than 40 years.^59^ We designed a new ZnGA-based catalyst by loading the main group metal salts onto the surface of ZnGA, and it was expected that the additional metal sites could enhance the performance of the catalyst. The resulting catalyst is named according to the type and amount of metal salt used, such as NaCl-100, which means that the type of metal salt used in the loading process is NaCl, and the proportion used is 1% of the ZnGA load. These catalysts supported with different metal were analyzed by powder X-ray diffraction (PXRD), Fourier transform infrared (FTIR) spectroscopy, and scanning electron microscopy (SEM) analysis (Figure 1). To verify the loading of the main group of metal salts, a series of supported catalysts were characterized using SEM and energy-dispersive X-ray (EDX) spectroscopy, compared with the simple blends of NaCl/ZnGA. As shown in Figure 1b, both catalyst systems consist of typical platelet-shaped particles. Combined with the SEM-EDS image of surface element distribution, we can find that the distribution of zinc and sodium in the supported catalyst coincides and uniformly distributes on the surface of the catalyst crystal, while the blends of NaCl/ZnGA showed contrary results (Figure 1b). The relevant SEM-EDS images of LiCl, MgCl_2_, and AlCl_3_ show similar conclusions, but the synthesis method was invalid in the production of the corresponding KCl–ZnGA catalyst (Figures S1–S4). Notably, when the loading of metal ions is fewer than 10^–2^ equiv of ZnGA, the metal ions cannot be detected via SEM energy-dispersive X-ray spectroscopy (SEM-EDS). These phenomena illustrate that we have successfully introduced additional species on the ZnGA surface; the uniform distribution is beneficial for the cooperation of ROP and ROCOP processes.

However, the distribution image of chlorine is not obvious in the above figure, due to the effect of the background carbon cloth. Therefore, we further characterized the structure of the catalyst, focusing on the form of sodium species on the surface of the catalyst. FTIR spectroscopic analysis shows typical peaks of ZnGA (Figure 1d). Meanwhile, according to the PXRD pattern of ZnGA, compared with the ZnGA pattern calculated by the crystal structure, the crystallinity of ZnGA was confirmed for the supported catalyst (Figure 1c). The introduction of metal ions does not cause any phase transitions or structural deformation of the crystal lattice, as shown in Figure 1c, so the PXRD patterns of different metal-supported ZnGA are similar. The supported metals are uniformly distributed on the catalyst surface and are positively correlated to the density of zinc distribution. The overall similar crystal structures demonstrate that the metal salts are mainly loaded on the surface of ZnGA. Combined with the decrease of the wide peak (3450 cm^–1^) in the FTIR image, it is believed that the metal salt is mainly reacted with the defect sites (hydroxyl groups, etc.) on the surface of ZnGA.

**Figure 2:**
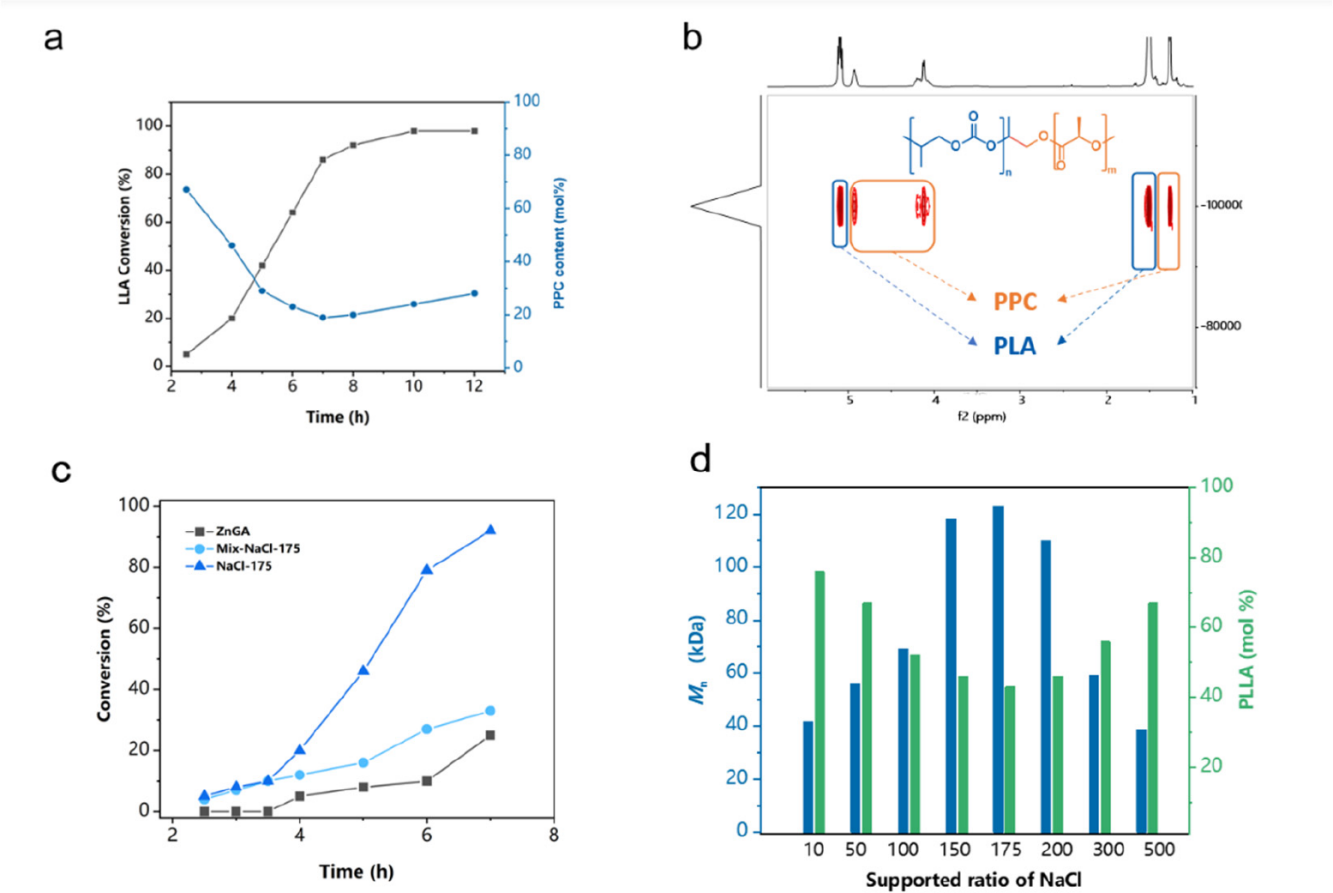
(a) Plots of the conversion of LLA and the PPC content. (b) Diffusion-ordered spectroscopy (DOSY) spectrum of the final polymers. NaCl-100:LLA:PO = 1:50:200, 2 MPa of CO_2_, the reaction was conducted in 1.5 mL of PO under 70 °C. (c) Kinetic plots for the polymerization of LLA by applying NaCl-175/NaCl-175 proportional blends/ZnGA as the catalyst in PO, at 70 °C, [LLA]/[Cat.] = 50:1. (d) The relationship between the NaCl supported amount and the molecular weight of the polymer obtained by the copolymerization/content of the PLLA segment (mol %), the reaction ratio was [PO]/[LLA]/[cat] = 200:20:1, at 70 °C, 12 h, and the CO_2_ pressure was 2 MPa.

The ZnGA with supported metal showed an excellent property more than the carriers in the cooperation of ROP and ROCOP processes. Firstly, we investigated the supported catalyst of NaCl-100 in the copolymerization of LLA, PO, and CO_2_. The ROP of LLA and ROCOP of PO/CO_2_ are in competition. As shown in Figure 2a, the CO_2_ and PO copolymerization is leading in the start, and LLA begins to polymerize after 2.5 h, as an induction period. Then, in the middle period of the whole copolymerization (2.5–8 h), the ROP of LLA shows obvious advantages in activity and provides more units in the resulted copolymer. When the conversion of LLA is near the end, the PO and CO_2_ copolymerization recovers. Diffusion NMR ordered spectroscopy (DOSY) was used to further confirm the segment structure of copolymers obtained by the copolymerization of LA, PO, and CO_2_. In the case of a copolymer, a single diffusion coefficient was expected, with all blocks expected to diffuse at the same rate, while, at the same time, the mixtures of homopolymers showed two coefficients.^60^ As shown in Figure 2b, we can find that the polymer obtained is a multiblock copolymer instead of a mixture of polylactide (PLA) and poly(propylene carbonate) (PPC).

The built-in structure provides more opportunity for the supported metal to cooperate with ZnGA than simply mixing metal salts and ZnGA in proportion. As shown in Figure 2c, we have compared the two catalyst systems in LLA polymerization, employing ZnGA independently showed the longest induction period. Adding NaCl with ZnGA or employing the supported catalyst could obviously shorten the induction period obviously. Notably, even after the induction period, the supported catalyst showed a further activity enhancement, which indicates that there is a stronger synergistic effect between ZnGA and the built-in metal center. Sodium has shown its excellent activity in the generation of alkoxide intermediates, which is beneficial for shortening the induction period. As we have reported, the initiation step was interpreted to be the insertion of epoxides into the M–Cl bond and the in-situ generation of alkoxide intermediates. Furthermore, this intermediate, as an active species, further initiated the ring-opening polymerization of cyclic ester.^61^ For further investigation of the induction period, we also designed a series of experiments (Table S2) by replacing the anion and initiator. As the results showed, the replacement of the anion had no effect on the induction period of LLA ROP (Table S2, entries 1–5). By contrast, the addition of mPEG200 or excess isopropanol could further shorten the induction period and lead to the production of polyether (Table S2, entries 6–10). For the heterogeneous catalyst systems, the ROP reaction should be propelled on the catalyst surface, and Na is active in ROP of LLA, as discussed in numerous relevant researches.^55^ Meanwhile, for the pure Na center, employing NaCl as catalyst, adding mPEG200 as an initiator showed no significant difference. So, we supposed that, in the induction period, the Na center is more active, because the alkoxide intermediate is linking too firmly on the Zn centers to attack the LLA monomers. When the growing chain is long enough or employing mPEG200 as an initiator, the polymerization rate increases as the linkages between the chain end and the Zn center becomes weaker. So, we prudently supposed that the built-in Na on the ZnGA surface could act as a more active center, and the special Na–Zn space structure and the fixed shortened distance between Na and Zn centers could contribute to an attractive heterometal coordination. After the induction period, the growing chain could leave the Zn center and transfer to the Na center more easily, which promotes the heterometal coordination and results in the further activity enhancement.

The substantive strengths of the supported catalysts are heterobimetallic synergy achieved by introducing new sites. Hence, in order to further study catalyst optimization and mechanistics, we have prepared a range of catalysts with different loadings of new metal sites. The introduction of Na center is benefit for the CO_2_-insert step, and the NaCl supported catalysts showed a decrease in selectivity, more than that of ZnGA, in terms of the selectivity of PPO (see Figure S6 and Table 1, entries 1–7). For the CO_2_-involved copolymerization and LLA ROP, the activity can be mainly demonstrated by the molecular weight. The results showed that, with the decrease in the loading, the molecular weight first increased and then decreased, while the molar percentage of LLA in the product decreased first and then increased (Figure 2d). NaCl-175 is the peak of both, i.e., *M*_n_ = 120.3 kDa, containing 43% PPLA segments. This phenomenon may be related to the competition of either ROP and ROCOP, or chain transfer and chain growth. For the aforementioned catalysts, it can be expected that the major parameters that influence both the ROP and ROCOP processes are the electrophilicity of the metal centers and the metal–metal distance. It is foreseeable that not all additional sites on the surface can be distributed appropriately and there is limit for the introduction of Na to turn into surface-active sites. When the content of Na increased (NaCl-500 to NaCl -175) first, the more active sites lead to higher activity and molecular weight of the resulting polymer. But for NaCl-175 to NaCl-10, the Na is introduced too excessively to hold an appropriated distance, the inordinate and crowded Na centers provide weakened heterobimetallic synergy. For various reasons, we preferred NaCl-175 as the optimal polymerization condition. Similar pattern was observed for other metal-supported catalysts (Table 1, entries 8–13), but the optimal loading ratio was not 175:1, probably due to the discrepancy in appropriated distances.

**Table 1:** LLA, CO_2_, and PO Copolymerization Catalyzed Using Different Metal Loading Catalysts^a^

entry	catalyst	[PO]/[LLA]/[cat]^b^	Con_LLA_^c^(%)	Sel_PPC_^d^(%)	PLA^e^(%)	PPO^f^ (%)	*M*_n_ (kDa)	*Đ*^g^
1	ZnGA	200:20:1	15	89	0	11	63.5	2.40
2	NaCl-10	200:20:1	94	95	76	3	41.9	2.19
3	NaCl-50	200:20:1	95	96	67	4	56.2	1.96
4	NaCl-150	200:20:1	96	95	46	4	136.9	1.81
5	NaCl-175	200:20:1	95	95	43	4	120.3	1.86
6	NaCl-300	200:20:1	94	94	56	5	59.3	2.33
7	NaCl-500	200:20:1	93	94	67	4	38.7	2.4
8	LiCl-125	200:20:1	56	92	44	6.5	113.3	1.60
9	LiCl-175	200:20:1	60	95	41	4.7	80.8	2.03
10	LiCl-225	200:20:1	70	94	48	3.8	65.1	2.20
11	MgCl_2_-125	200:20:1	19	96	6	3	88.7	1.87
12	MgCl_2_-175	200:20:1	8	98	16	3	123.0	1.60
13	MgCl_2_-225	200:20:1	25	99	17	2.1	99.4	1.70

a The different amounts of metal-supported catalysts were added in 1.5 mL of PO in a 25-mL autoclave. The autoclave was pressurized with CO_2_ to 2.0 MPa and then was heated to 70 °C, which continued for 12 h.

b Molar ratio.

c The results determined by the conversion of PO/LLA in the crude copolymerization mixture by ^1^H NMR analysis.

d Selectivity for cPC over PPC, determined by ^1^H NMR analysis.

e Selectivity for PPO over polyester, determined by ^1^H NMR analysis.

f The amount of PPC in the polymer obtained by sedimentation, determined by ^1^H NMR analysis.

g Determined by GPC using CHCl_2_ as the solution, calibrated with polystyrene standard.

To further understand the influence of the additional metals on the ZnGA surface, the copolymerization of PO and CO_2_ were studied (Table 2, entries 1–5). In the alternating copolymerization of CO_2_ and PO, the introduction of the main group metals is selective for the CO_2_-insert step, which leads to the reduction of polyether, and the molecular weight showed an obvious increase. Under the same polymerization condition ([PO]/[cat] = 200:1, 12 h), the PPO selectivity of MgCl_2_-100 decreased from 16% to 5%, compared with the pure ZnGA. The introduction of alkali metals Li and Na could also weaken PPO selectivity, both of which decreased to ∼16% from 10% (Li) and 9% (Na). Meanwhile, the introduction of Al did not bring any selectivity increase for PPC. In terms of molecular weight, the introduction of Na and Al brought immense enhancement, presenting copolymers with molecular weights of 206.3 and 216.4 kDa. Respectively, other metals also promoted the copolymerization in molecular weight, as shown in Table 2.

**Table 2:** LLA, CO_2_, and PO Copolymerization Catalyzed Using Different Metal-Supported Catalysts^a^

entry	catalyst	[PO]/[LLA]/[cat]^b^	Con^c^(%)	Sel_cPC_^d^(%)	Sel_PPO_^e^(%)	PPC^f^(%)	*M*_n_ (kDa)	*Đ*^g^
1	ZnGA	200:0:1	47	5	16	83	125.7	1.73
2	MgCl_2_-100	200: 0:1	62	4	5	97	153.3	1.51
3	NaCl-100	200:0:1	62	4	9	94	206.3	1.33
4	LiCl-100	200:0:1	64	4	10	94	133.6	1.36
5	AlCl_3_-100	200:0:1	71	6	19	82	216.4	1.72
6	ZnGA	200:20:1	15	1	11	89	63.5	2.40
7	MgCl_2_-100	200:20:1	-	1	1	99	55.8	1.94
8	NaCl-100	200:20:1	70	1	5	62	43.5	2.40
9	LiCl-100	200:20:1	70	1	6	40	95.9	1.71
10	AlCl_3_-100	200:20:1	30	1	9	14	92.4	1.92

a The different amounts of metal-supported catalysts were added in 1.5 mL of PO in a 25-mL autoclave. The autoclave was pressurized with CO_2_ to 2.0 MPa and then was heated to 70 °C, which was continued for 12 h.

b Molar ratio.

c Entries 1–5 show the conversion of PO, and entries 6–10 show the conversion of LLA. The results determined by the conversion of PO/LLA in the crude copolymerization mixture by ^1^H NMR analysis.

d Selectivity for cPC over PPC, determined by ^1^H NMR analysis.

e Selectivity for PPO over polyester, determined by ^1^H NMR analysis.

f The amount of PPC in the polymer obtained by sedimentation, determined by ^1^H NMR analysis.

g Determined by GPC using CHCl_2_ as the solution, calibrated with polystyrene standard.

As supposed, different additional sites lead to diverse products in the copolymerization of LLA, PO, and CO_2_. (See Table 2, entries 6–10.) For the pure ZnGA, although a low conversion of LLA was confirmed, we detected no PLLA ingredient after sedimentation, which indicated that the LLA monomer was transferred into PLLA oligomer independent of the copolymerization of PO and CO_2_. (See Table 2, entry 6.) The introduction of Na, Li, and Al is helpful for catalyzing the ROP of LLA and ROCOP of PO/CO_2_ simultaneously, and the corresponding terpolymers of PLLA and PPC units were obtained. The PLLA content in polymers is different due to different activity and selectivity of built-in metal species, the content decreases according to the order of Na > Li > Al (Table 2, entries 8–10). Notably, the introduction of Mg completely inhibits the ROP of LLA and the continuous ROP of PO, so no significant PLLA and PPO units were observed by ^1^ H NMR analysis.

**Figure 3:**
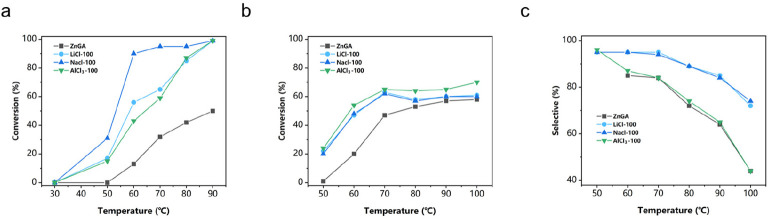
(a) Effect of temperature on activity, using different metal-supported catalysts ([LLA]/[Cat.] = 200:1), at 70 °C, 2 MPa CO_2_, 12 h. (b) Effect of temperature on activity. (c) Effect of temperature on selectivity, using different metal-supported catalysts ([PO]/[Cat.] = 200:1), at 70 °C, 2 MPa CO_2_, 12 h.

Although most heterogeneous catalysts are stable under high temperature, it is important for the catalyst to be active enough under low reaction temperature for the economic energy demand of industrial application. We have studied the effect of reaction temperature for ZnGA, NaCl-100, LiCl-100, and AlCl_3_-100 in LLA polymerization (see Figure 3a). The results showed that the introduction of surface heterometallic sites significantly increased the activity of LLA ROP; especially at 50 °C, ZnGA basically lost its polymerization activity, but the supported catalyst could still catalyze the reaction. The type of additional metal site is also associated with the activity of LLA ROP. With higher temperatures, the introduction of Na demonstrated the best activity therein. For instance, the conversion of NaCl-100 reached 93% at 60 °C, while the conversion rate of the rest of the supported catalysts was only ∼45%. In terms of selectivity, the introduction of the main group metal sites did not bring a significant difference, and the resulting PLLA product contained <1% of the polyether units observed by ^1^ H NMR.

The selectivity and activity for ZnGA, NaCl-100, LiCl-100, and AlCl_3_-100 versus temperature is shown in Figures 3b and 3c. Activity disparity between these catalysts was even more obvious at the lower temperatures. At 50 °C, metal-supported catalysts could convert 23% PO to PPC while ZnGA lost activity completely. At 60 °C, the built-in structure brought nearly three times more activity than ZnGA (Figure 3b). As the temperature increases, the conversion rate of PO began to maintain equilibrium, because of the increase in viscosity of the polymerization system, and the side reactions increased. NaCl-100 and LiCl-100 showed better selectivity than the others at high temperatures (Figure 3c). When the temperature achieved 80 °C, the selectivity of alkali-metal-supported catalysts was maintained at 90% and simultaneously the selectivity of ZnGA and AlCl_3_-100 decreased quickly, due to severe backbiting reactions. The above phenomenon revealed that the strong synergistic effect between ZnGA and the built-in metal center (alkali metal) can bring higher activity and selectivity to the alternating copolymerization of PO and CO_2_.

**Figure 4:**
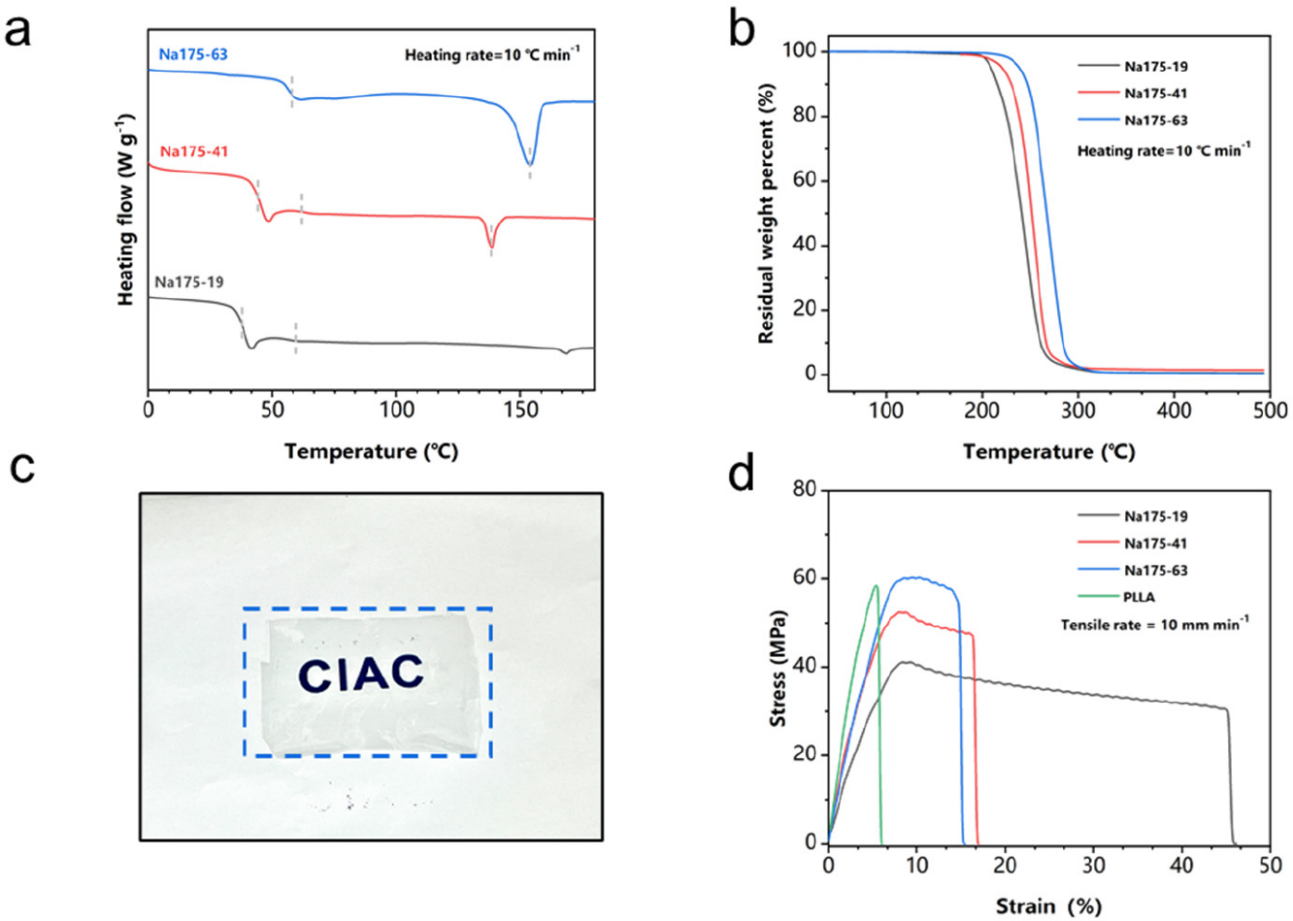
(a) DSC test curves of CO_2_-based copolymers with different content of the PLLA segment. (b) TGA curves of CO_2_-based copolymers with different content of the PLLA segment. (c) Material transmittance and spline display. (d) Representative stress–strain curves of PLLA and CO_2_-based copolymers.

After the establishment of the optimal catalytic systems, a series of PPC–PLLA random copolymers with PLLA content of 19–91 mol % was prepared. The PPC content of the terpolymers was adjusted by shifting co-catalysts, CO_2_ pressure, and temperature by which the regulation of thermodynamic performance would be reached. As listed in Table S3, all reactions showed activity and the conversions of LLA were >95%. All the obtained terpolymers had high molar masses (*M*_n_ > 100 kDa, and the wider distributions might be attributed to the transesterification at the interface of heterogeneous catalysts) and exhibited better *T*_g_ than PPC. As shown in Figure 4a, their glass-transition temperature (*T*_g_) values vary between 40 °C for alternating poly(propylene carbonate) and 65 °C for the PLA. With the increase of PLA content (from 19% to 63%), the decomposition temperature with 5% weight loss (*T*_d,5%_) of the terpolymers increased from 209 °C to 240 °C (see Figure 4b). Only one *T*_g_ is detected for sample NaCl175-19 with a lower PLLA content. All the other terpolymers with higher PLLA content exhibit a melting temperature (*T*_m_) in the range of 135–150 °C, indicating that phase separation between polycarbonate and polyester blocks occurs. With the decrease of PLLA segments (from Na175-41 to Na175-19), the melting regions disappeared, indicating a transformation from semicrystalline to amorphous polymer. Thus, the solvent-cast film of Na175-19 turned out to display excellent transmittance (Figure 4c).

With the introduction of rigid segments (PLLA), these terpolymers has demonstrated strong tensile strength (all >40 MPa), improved elongation (45% vs 7%), excellent transmittance, and low water vapor permeability (1.7 × 10^–11^ g m^–1^ s^–1^ Pa^–1^), which prospectively leads to its application in the packaging and other fields (Figure 4d). However, compared with the materials obtained by Feng and Athanassiou, the copolymers prepared showed higher tensile strength obtained by the expense of partial loss of elongation.^47,62^ Compared with sufficiently reasonable distribution of segments in PU, the segment structure of the CO_2_-based copolymer results in the suboptimal performance, which demonstrated the imperfect complementarity of the rigid and flexible segments. Considering the relatively high tensile strength, better elongation than PLLA, and high light transmittance of Na175-19 copolymer, the water vapor permeability rate of Na175-19 was characterized. This material exhibits a better water vapor permeability rate (1.7 × 10^–11^ g m^–1^ s^–1^ Pa^–1^), compared to that reported by Athanassiou et al. (3.0 × 10^–11^ g m^–1^ s^–1^ Pa^–1^).

**Figure 5:**
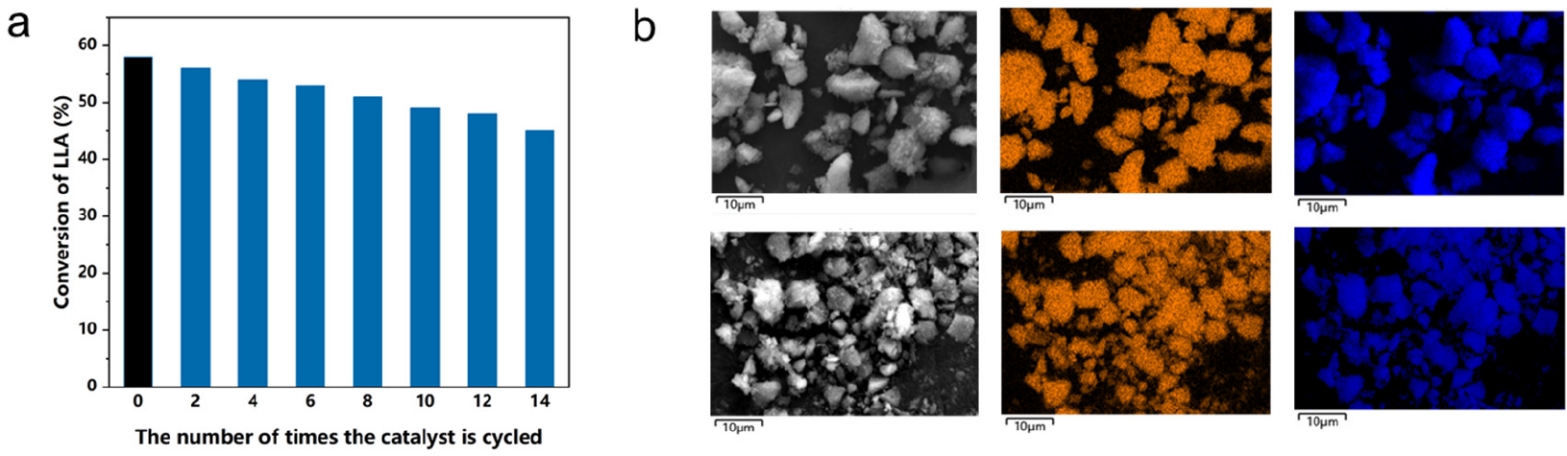
(a) Schematic diagram of the number of catalyst cycles and the corresponding LLA conversion, as NaCl-100 in PO, at 70 °C, [LLA]/[PO] = 50:1. (b) SEM-EDS images of unused NaCl-100 vs NaCl-100 cycled 14 times. (NaCl-100 above, zinc in yellow, sodium in blue).

Adhering the principles of green chemistry and atom economy, the recycling of catalysts is of great significance, which can not only reduce the metal residue in polymers but also increase competitiveness through lower production costs. The supported ZnGA catalysts can be conveniently separated by simple techniques (filtration, centrifugation, etc.) for recycling. For this advantage, we combined with SEM-EDS, taking NaCl-100 as the research object to explore the morphology and surface element distribution changes of the catalyst after multiple polymerizations. According to Figure 5a, the activity of catalyst recovered in air could still be maintained after 14 cycles, which further proved the remarkable moisture- and air-stable nature of this type of catalyst. As the number of catalytic cycles increases, however, the morphology of the catalyst is no longer the standard platelet-like crystal, and small fragments begin to appear.^63^ In addition, the metal content of the surface is reduced at the same time, which coincides with the gradually decreasing conversion (Figure 5b).

## Conclusion

We prepared a wide range of main-group metal-supported ZnGA samples, which were analyzed by PXRD, FTIR, and SEM-EDS. The results demonstrate that the crystal structure of ZnGA remains while the additional metal could react with the defect sites (hydroxyl groups, etc.) to form new active sites. The catalytic system can be applied in the copolymerization of CO_2_ and PO and the polymerization of LA. Compared with ZnGA and similar blends of ZnGA/main group metal salts, the supported catalyst with the additional sites on the surface especially Na, showed higher activity and selectivity from heterobimetallic synergy, which attained the terpolymerization. Notably, even after recovery in air, the activity of NaCl-100 could still be maintained after 14 cycles. Meanwhile, we studied the activity and selectivity affected by the additional metal site loading and the temperature, and optimal catalytic conditions were determined. A wide range of terpolymers were obtained by adjusting the content of PPC. These materials exhibit improved properties, especially in water resistance and light transmission, which proved the feasibility of the design concept for the complementarity of the rigid and flexible segments. Subsequently, we intend to further optimize the catalytic system to develop more-suitable material in packaging and other fields.

## Supporting Information Available

Experimental details, tables of polymerization data, SEM-EDS patterns (PDF)
