# Supplementary material for: Sustainable, Recyclable, and Bench-Stable Catalytic System for Synthesis of Poly(ester-b-carbonate)
Source: Chem Bio Eng. 2024 Jun 3;1(6):559–67. doi: 10.1021/cbe.4c00064 (PMC11835286; doi:10.1021/cbe.4c00064)
Supplement: Supplementary file 1 — be4c00064_si_001.pdf [file be4c00064_si_001.pdf]

SUPPORTING INFORMATION

Sustainable, Recyclable and Bench-Stable Catalytic System for Synthesis of Poly(ester-b-carbonate)

Yifan Jia[1,2], Bokun Li[1,2], Yifei Sun[1,2], Chenyang Hu[1], Xiang Li[1], Shunjie Liu[1], Xianhong Wang[1], Xuan Pang[1,2], Xuesi Chen[1,2]**

[1]Key Laboratory of Polymer Ecomaterials, Changchun Institute of Applied Chemistry, Chinese Academy of Sciences, 5625 Renmin Street, Changchun 130022, P. R. China

[2]School of Applied Chemistry and Engineering, University of Science and Technology of China, Hefei 230026, P. R. China

Contents

1. Materials and characterization

2. Synthesis and recycle of ZnGA and supported catalysts

3. Characterization of ZnGA and supported catalysts

4. Copolymerization of PO, CO_2 and LLA

5. References

1. Materials and characterization

General

Starting materials and solvents for the synthesis of complexes were purchased from Aldrich Inc. and used without further purification. Propylene oxide was distilled from CaH_2 under argon atmosphere, CO_2 (99.95%) was purchased from Siping Jianxin Gas Company and used as received. NMR spectra were recorded on Bruker AV 500 M in $CDCl_3$. Chemical shifts were given in parts per million from TMS. Gel permeation chromatography (GPC) measurements were conducted with a Waters 515 GPC with $CHCl_3$ as eluent vs. polystyrene standards.

Copolymerization

All copolymerization were performed in 25 mL steel autoclaves. The autoclaves were heated to 110℃ for 12 h, then cooled down to room temperature in glovebox prior to use. In a typical polymerization experiment, catalysts, cocatalyst and PO in desired ratio were added into an autoclave containing a magnetic bar. The autoclave was sealed and filled with CO_2, heated to the desired temperature. When the reaction reached certain reaction time, CO_2 was released slowly, and a sample of crude reaction mixture was taken for NMR test. The polymer was dissolved with CH_2Cl_2 and isolated by precipitation with acidified ethanol three times. The polymer was then dried under vacuum at 40 °C for 24 h.

The glass transition temperature (T_g) was determined by differential scanning calorimeter (DSC, Netzsch Model 204) with a heating and a cooling rate of 10 °C/min from 10 °C to 200 °C under nitrogen atmosphere. Notably, the T_g values were adopted from the second heating curves. TGA measurements were performed in a Perlin Elmer Pyris Diamond TG/DTA analyzer under nitrogen atmosphere at a heating rate of 10 °C/min in the temperature range of 30-500 °C.

The tensile mechanical test was performed using a WSM-20kN universal testing machine. The dumbbell-shaped specimens were processed from injection molding using Minijet Pro D-76227 Karlsruhe injection machine for ISO 527-2 type 5B. The injection conditions were as follows: the barrel temperature: 160 ~ 170 ^{o}C, mold temperature: 40 ~ 45 ^{o}C, and injection pressure: 200 ~ 450 MPa. Uniaxial extension experiments (20 mm min^{-1} cross-head speed) were run according to ISO 527.[1]

The water vapor transmission rate (WVTR) and water vapor permeability (WVP) of the PLAPC films were determined gravimetrically according to the ASTM E96 standard test method.[2, 3] The investigation was carried out at ambient temperature, under 100% relative humidity gradient (ΔRH%). Deionized water (400 μL) (which generates 100% RH inside a permeation test cell) was added into each such cell of given dimensions (internal

diameter of 7 mm, inner depth of 10 mm). The films were cut into circles with a cutting press and put on top of the permeation test cells. The cells were placed inside a desiccator containing anhydrous silica gel, which served as a desiccant for maintaining 0% RH. The water vapor transferred through the film and absorbed by the desiccant was determined from the change in weight of the cells at hourly intervals over an 8 h period. A set of electronic scales (accuracy of ±0.1 mg) was employed to record loss in weight over time, and these values were plotted as a function of time. The WVTR was determined from the slope of each line obtained from linear regression according to the following equation

$$\mathrm{WVTR}(\mathrm{g}/m^2) = \frac{\mathrm{slope}}{\text{area of the film}}$$

WVP measurements were replicated three times for each PLA/PPC film and their composites with CCM. Values for WVP were calculated by the following equation

$$\mathrm{WVTR}(\mathrm{g/m^2 \cdot day \cdot Pa}) = \frac{\mathrm{WVTR} \times \mathrm{l} \times 100}{\mathrm{P_s} \times \mathrm{RH}}$$

where l (m) is the mean film thickness measured with a micrometer (accuracy of 0.001 mm), ΔRH (%) is the relative humidity gradient percentage, and PS (Pa) denotes WVP saturation at 25 °C.

2. Synthesis and recycle of ZnGA and supported catalysts

Synthesis of ZnGA and supported catalysts

Referring to the literature, glutaric acid and zinc oxide were stirred in toluene at 70 ℃ for 12h to obtain zinc glutarate catalyst, and then using tetrahydrofuran (THF) as a medium the corresponding proportion of metal salts was added and stirred in a water bath environment of 40 ℃ for 12h to achieve uniform loading, and the obtained solid was washed 3 times by anhydrous THF and acetone, and then placed in a vacuum oven for 24h to obtain supported zinc glutarate catalysts.[4]

Recycle of ZnGA and supported catalysts

The reaction sample was dissolved with an appropriate amount of CH2Cl2, the catalyst was recovered by centrifugation technology, and the precipitate obtained by centrifugation was washed with CH_2Cl_2 for 3 times. After the catalyst was recovered, the reaction temperature was 70°C, and the reaction ratio was [PO]/[LLA]/[cat]= 200:20:1, and it was carried out in a 25 ml steel autoclaves.

3. Characterization of ZnGA and supported catalysts

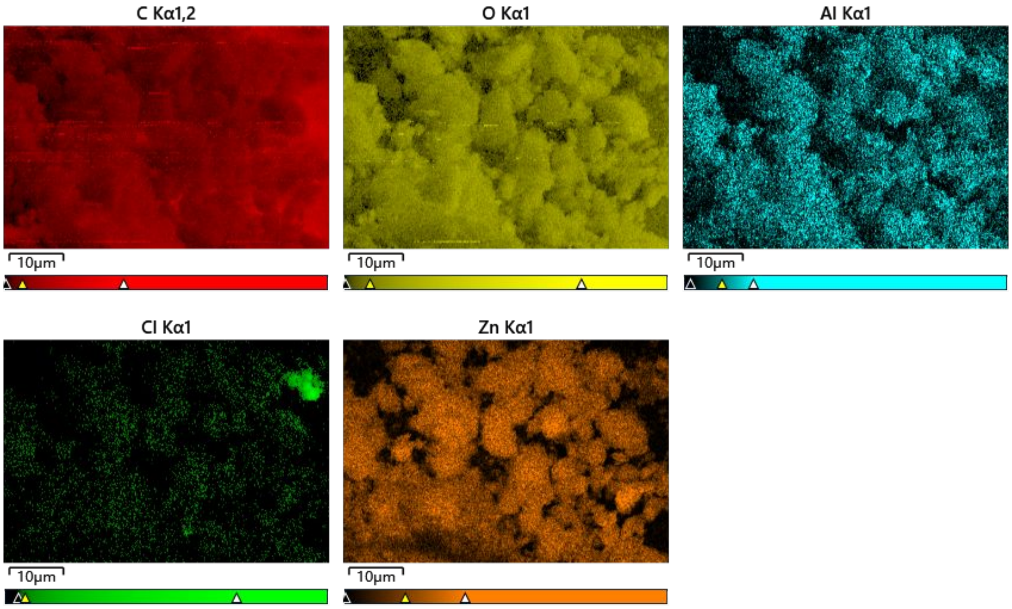

Figure S1. SEM-EDS image of $AlCl_3$-100

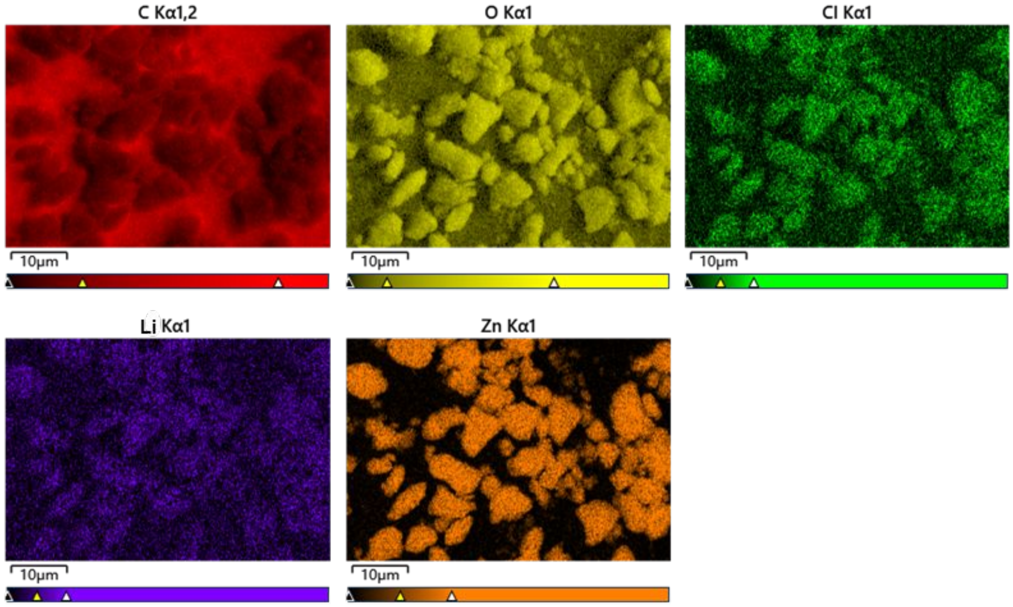

Figure S2. SEM-EDS image of LiCl-100

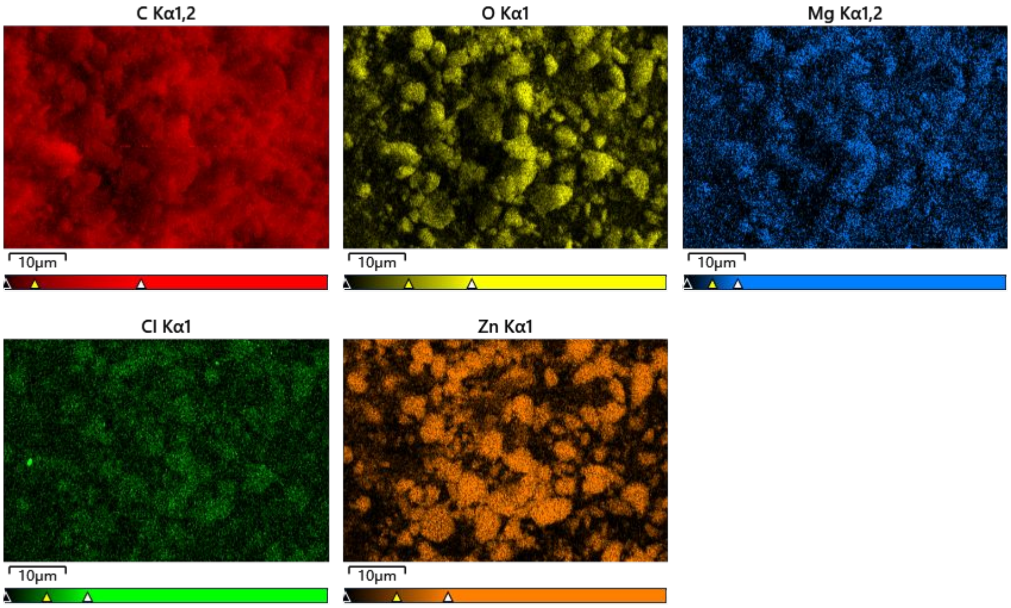

Figure S3. SEM-EDS image of $MgCl_2$-100

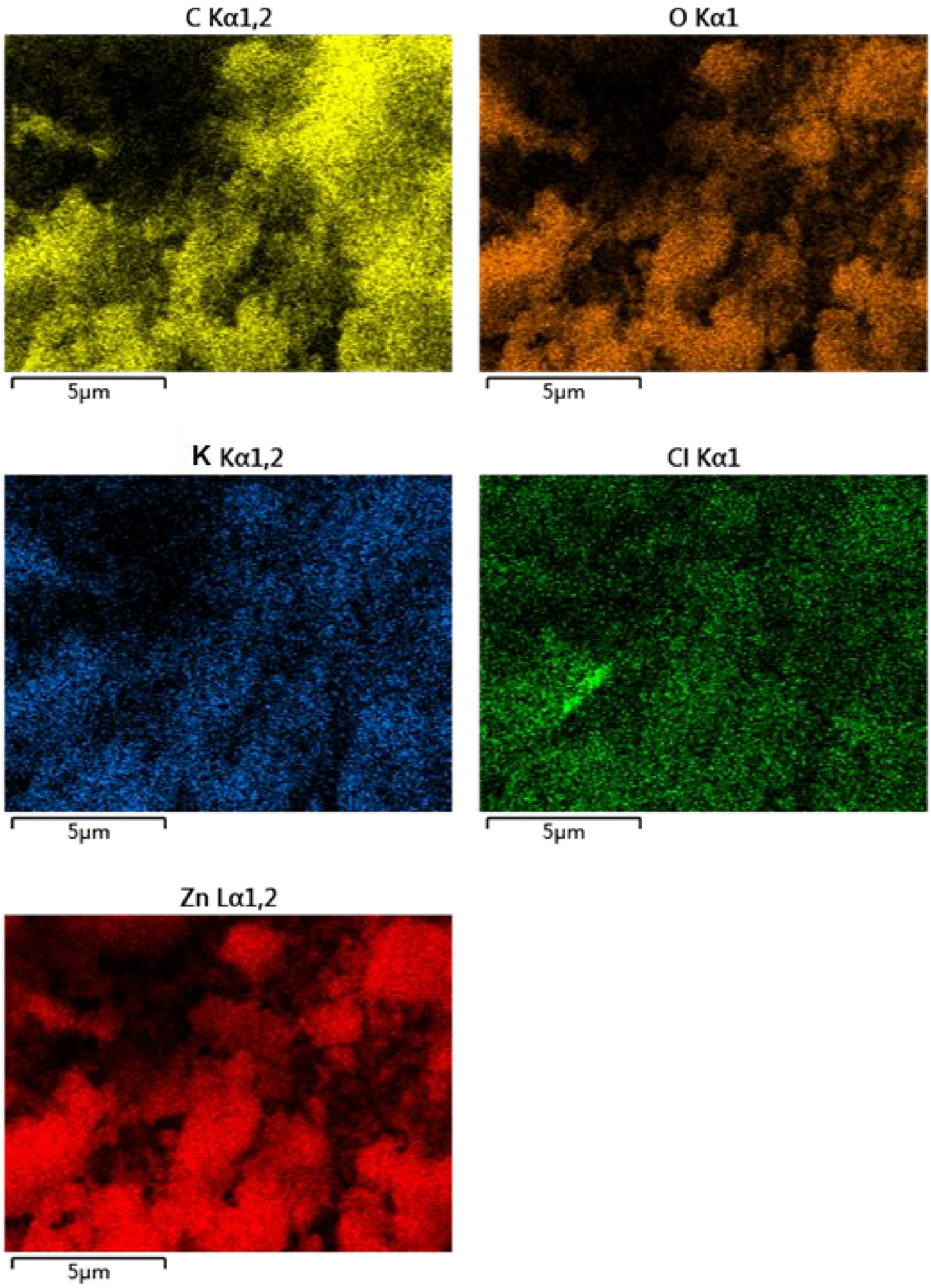

Figure S4. SEM-EDS image of KCl-100

Table S1 the EDS data of ahead ZnGA/MCl

AlCl3-100 EDS Data			LiCl-100 EDS Data		
**Element**	**Wt%**	**At%**	**Element**	**Wt%**	**At%**
C	55.06	70.4	C	56.77	70.4
O	26.79	25.2	O	24.6	24.39
Al	0.28	0.16	Li	2.01	1.32
Zn	17.68	4.16	Zn	16.44	3.81
Cl	0.19	0.08	Cl	0.18	0.08

MgCl2-100 EDS Data			NaCl-100 EDS Data		
**Element**	**Wt%**	**At%**	**Element**	**Wt%**	**At%**
C	58.5	72.27	C	58.6	72.44
O	26.02	24.13	O	24.9	23.11
Mg	0.16	0.1	Na	1.64	1.06
Zn	15.18	3.44	Zn	14.75	3.35
Cl	0.14	0.06	Cl	0.1	0.04

KCl-100 EDS Data

Element	Wt%	At%
C	64.13	75.64
O	23.89	22.07
K	0	0
Zn	10.41	2.26
Cl	0.07	0.03

4. Copolymerization of PO, CO_2 and LLA

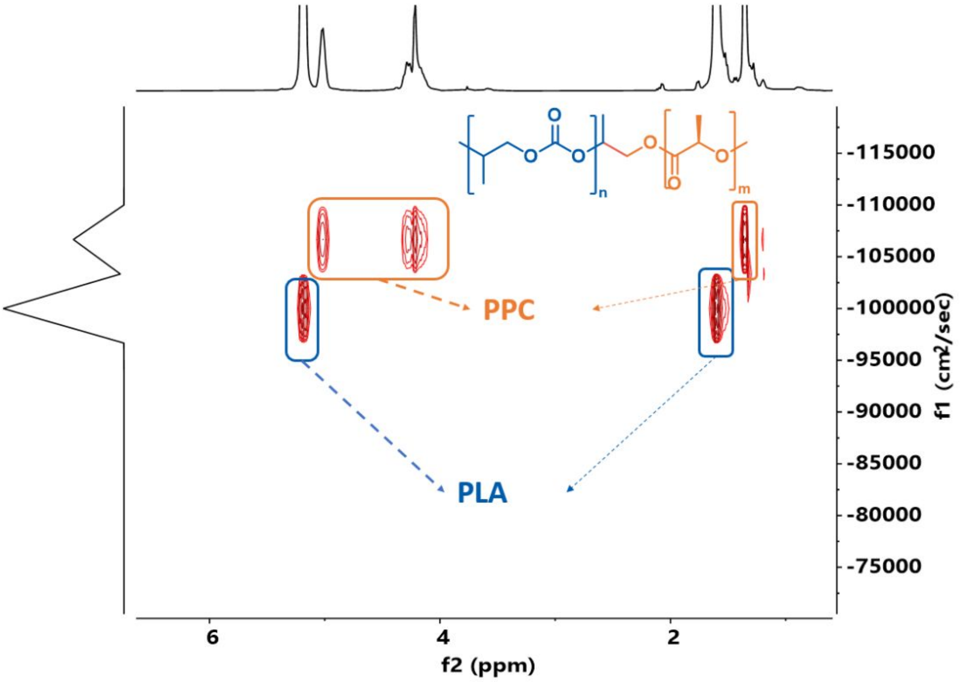

Figure S5. The diffusion-ordered spectroscopy (DOSY) spectrum of the mixed polymers

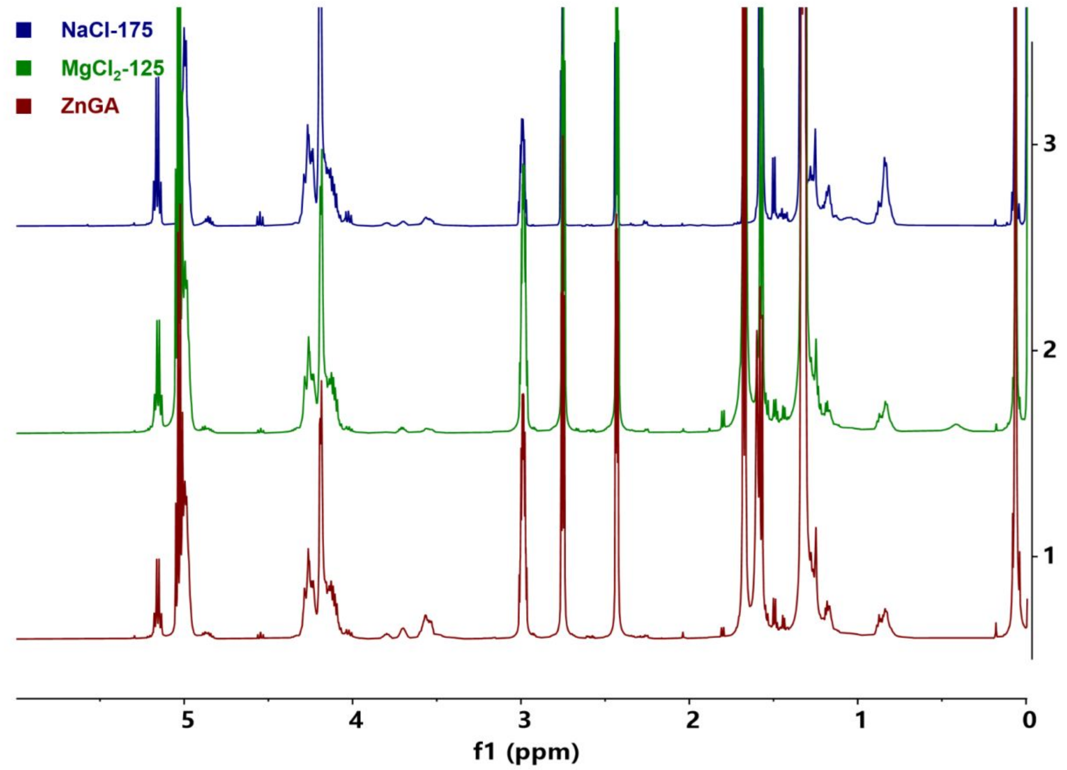

Figure S6. The ^{1}H NMR spectrum of the Conversion of LA/PO/CO_2 catalyzed by NaCl-175/$MgCl_2$-125/ZnGA, using different metal supported catalysts ([PO]/[LLA]/[Cat.] = 200: 20: 1), at 70 °C, 2 MPa CO_2, 12 h.

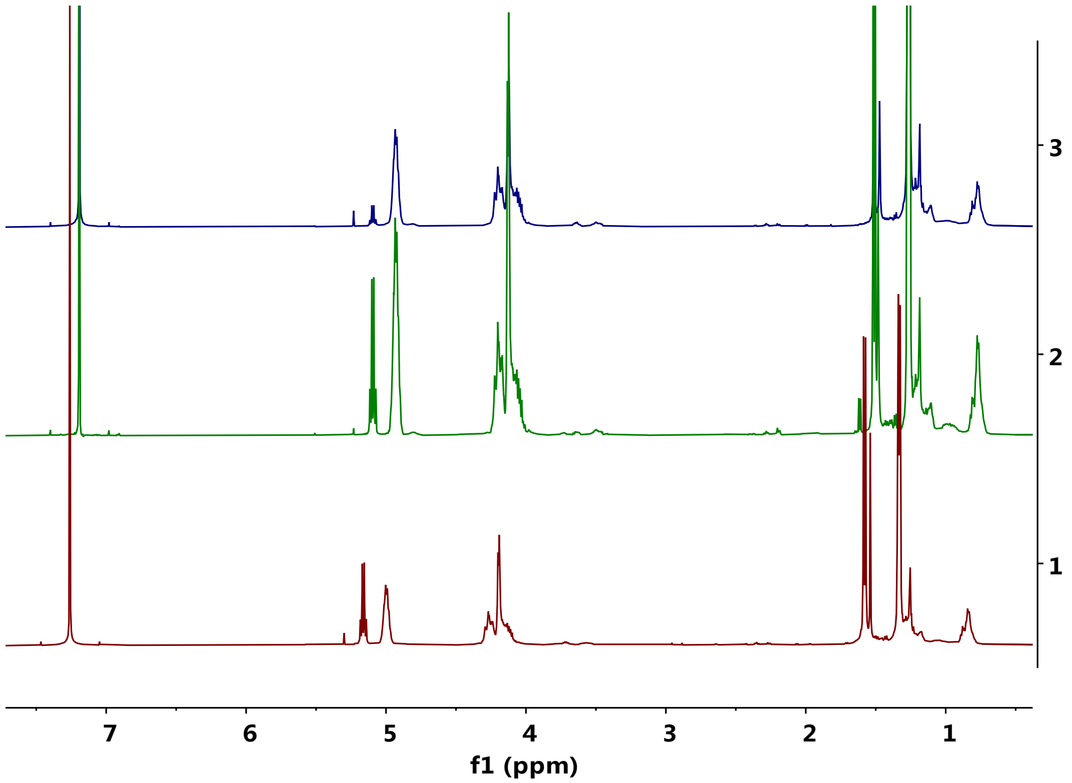

Figure S7. The ^{1}H NMR spectrum of NaCl-19/41/63

Table S2 The induction period of LLA polymerization catalyzed using different catalysts and cocatalysts[a]

Entry	Cat.	Initiators	[PO]/[LLA]/[cat]/[cocat.][b]	t (h)	Con. [c] (%)
1	NaCl-175	-	200:20:1	2.5	5
2	NaF-175	-	200:20:1	2.5	4
3	NaBr-175	-	200:20:1	2.5	5
4	Mix-NaF-175	-	200:20:1	2.5	4
5	Mix-NaBr-175	-	200:20:1	2.5	5
6	ZnGA	mPEG-200	200:20:1:1	1	5
7	ZnGA	IPA	200:20:1:1	1.5	4
8	ZnGA	mPEG-200	200:20:1:1	2.5	67
9	NaCl-175	mPEG-200	200:20:1:1	1	6
10	NaCl-175	IPA	200:20:1:1	1.5	6

a The different metal-supported catalysts were added in 2 mL PO in a 25 mL autoclave, and then was heated up to 70 °C, continue 12 h.

b Molar ratio.

c the conversion of LLA. The results determined by the conversion of LLA in the crude copolymerization mixture by ^{1}H NMR analysis.

Table S3 The LLA, CO2, and PO copolymerization catalyzed using different catalysts and initiators [a]

Entry	Cat.	[PO]/[LLA]/[cat][b]	Con. [c] (%)	Sel.$_{PPC}$ [d] (%)	PLA [e] (%)	PPO [f] (%)	M_n [g] (kDa)	Đ[g]
1	NaCl-175	200:20:1	99	96	19	3	136.9	1.52
2	NaCl-175	200:40:1	99	96	41	1	123.3	1.60
3	NaCl-175	200:60:1	99	95	63	1	108.8	1.63
4 [h]	NaCl-100	200:60:1	80	95	80	1	108.8	1.63
5[i]	NaCl-175	200:20:1	99	96	19	3	136.9	1.52
6	NaCl-175/PPNCl	200:20:1	70	94	48	3.8	65.1	2.20
7	NaCl-175/mPEG200	200:20:1	19	96	6	3	88.7	1.87

a The different amounts of metal-supported catalysts were added in 2 mL PO in a 25 mL autoclave. The autoclave was pressurized with CO_2 to 2.0 MPa and then was heated up to 70 °C, continue 24 h.

b Molar ratio.

c the conversion of LLA. The results determined by the conversion of PO/LLA in the crude copolymerization mixture by ^{1}H NMR analysis.

d Selectivity for PPC over Polyester, determined by ^{1}H NMR analysis.

e The amount of PLA in the polymer obtained by sedimentation, determined by ^{1}H NMR analysis. f The amount of PPO in the polymer obtained by sedimentation, determined by ^{1}H NMR analysis. g Determined by GPC using $CHCl_2$ as the solution, calibrated with polystyrene standard. h similar to a, except to heat continue 12 h. I The autoclave was pressurized with CO_2 to 2.0 MPa after reacting 4h.

5. References

(1) Yang, Z.; Hu, C.; Pang, X.; Chen, X. Sequence Design in Terpolymerization of E-Caprolactone, Co2 and Cyclohexane Oxide: Random Ester-Carbonate Distributions Lead to Large-Span Tunability. *Chin. Chem. Lett.* **2024**, *35*, 109340.

(2) Ho, T. T. T.; Zimmermann, T.; Ohr, S.; Caseri, W. R. Composites of Cationic Nanofibrillated Cellulose and Layered Silicates: Water Vapor Barrier and Mechanical Properties. *ACS Appl. Mater. Interfaces* **2012**, *4*, 4832-4840.

(3) Tran, T. N.; Paul, U.; Heredia-Guerrero, J. A.; Liakos, I.; Marras, S.; Scarpellini, A.; Ayadi, F.; Athanassiou, A.; Bayer, I. S. Transparent and Flexible Amorphous Cellulose-Acrylic Hybrids. *Chem. Eng. J.* **2016**, *287*, 196-204.

(4) Ang, R.-R.; Sin, L. T.; Bee, S.-T.; Tee, T.-T.; Kadhum, A. A. H.; Rahmat, A. R.; Wasmi, B. A. Determination of Zinc Glutarate Complexes Synthesis Factors Affecting Production of Propylene Carbonate from Carbon Dioxide and Propylene Oxide. *Chem. Eng. J.* **2017**, *327*, 120-127.
